# Deletion of the E3 ubiquitin ligase LRSAM1 fosters intracellular *Staphylococcus aureus* survival

**DOI:** 10.3389/fcimb.2025.1597830

**Published:** 2025-08-11

**Authors:** Ole Plöhn, Abhishek Kumar Singh, Clara Greger, Hannes Wolfgramm, Madina Baglanova, Kristin Surmann, Uwe Völker, Barbara M. Bröker, Karsten Becker, Ulrike Seifert, Clemens Cammann

**Affiliations:** ^1^ Friedrich Loeffler - Institute of Medical Microbiology, University Medicine Greifswald, Greifswald, Germany; ^2^ Department of Functional Genomics, Interfaculty Institute for Genetics and Functional Genomics, University Medicine Greifswald, Greifswald, Germany; ^3^ Institute of Immunology, University Medicine Greifswald, Greifswald, Germany

**Keywords:** *S. aureus*, ubiquitin, selective autophagy, LRSAM1, intracellular bacteria, E3 ligase

## Abstract

**Background:**

Intracellular invasion and persistence of *Staphylococcus aureus* can lead to chronic infection and is an effective strategy for the pathogen to evade the host immune response and antibiotic therapy. Selective ubiquitination of bacterial surfaces via E3 ubiquitin ligases is a mechanism by which host cells combat intracellular bacteria and target them for autophagosomal degradation. However, knowledge of the E3 ligases involved in intracellular recognition of *S. aureus* is still very limited.

**Methods:**

We studied A549 lung epithelial cells during *S. aureus* infection, focusing on the role of the E3 ligase leucine rich repeat and sterile alpha motif containing 1 (LRSAM1). We used the CRISPR-Cas9 system to generate LRSAM1-deficient A549 cells and monitored intracellular bacterial survival, activation of host cellular signalling pathways related to cytokine production, and host cell death during *S. aureus* infection.

**Results:**

In LRSAM1-deficient host cells we observed a significant increase in intracellular bacterial load, which was accompanied by an increased host cell death and elevated secretion of the pro-inflammatory cytokine IL-6. Despite induced selective autophagy, LRSAM1 knockout host cells were incapable of lowering and eliminating the pathogen, which seems to be caused by the reduced ubiquitination of the bacterial surface.

**Conclusion:**

The results indicate a significant role of LRSAM1 in the clearance of intracellular *S. aureus*. This contributes to a deeper understanding of the host cellular responses to *S. aureus* infection and will facilitate the development of novel therapeutic strategies to combat intracellularly persistent *S. aureus*.

## 1 Introduction

The Gram-positive bacterium *Staphylococcus aureus* (*S. aureus*) is an opportunistic pathogen responsible for a diverse spectrum of diseases, ranging from minor skin and wound infections to life-threatening systemic disturbances such as bacteremia, septic arthritis, and endocarditis (Tong et al., 2015). In addition, *S. aureus* is a leading cause of hospital-acquired pneumonia, particularly in intensive care settings, where it is associated with high morbidity and mortality (DeRyke et al., 2005; Kalil et al., 2016; Torres et al., 2017). Furthermore, influenza virus infection can predispose individuals to secondary *S. aureus* pneumonia, resulting in severe coinfections that are characterized by rapid clinical deterioration and poor outcomes (Morris et al., 2017). Its pathogenicity is based on an extensive, often redundant and multifunctional arsenal of virulence factors as part of the core or accessory genome (Cheung et al., 2021). Moreover, the pathogen has evolved a variety of strategies to evade immune detection and clearance (de Jong et al., 2019). An effective strategy for this is to switch to the small-colony variant (SCV) phenotype, which is particularly adapted to an intracellular lifestyle and may lead to persistent and relapsing *S. aureus* infections that are often refractory to antibiotic therapy (Proctor et al., 2006). Moreover, the treatment of *S. aureus* infections has generally become increasingly challenging due to the emergence of antibiotic-resistant strains, in particular methicillin-resistant *S. aureus* (MRSA) clonal lineages, which compromise the efficacy of conventional antibiotic therapies (Lee et al., 2018).

One mechanism to evade the host immune response is the intracellular survival and persistence of *S. aureus* in epithelial cells and phagocytes (Strobel et al., 2016; Rodrigues Lopes et al., 2022). Elimination of intracellular pathogens relies on host cellular defense mechanisms such as pathogen recognition, ubiquitination by host E3 ligases, and subsequent degradation through selective autophagy. Selective autophagy is a fundamental biological process that targets specific cellular components for degradation in intracellular vesicles, thereby, facilitating cellular homeostasis, efficient recycling of cellular constituents, and adaptation to stress conditions. In the context of infection, studies have demonstrated that selective autophagy plays a pivotal role in the host’s defense against intracellular pathogens, including bacteria, viruses, and parasites (Gomes and Dikic, 2014; Ponpuak et al., 2010; Shoji-Kawata and Levine, 2009; Besteiro, 2019). The recognition of intracellular bacteria for the autophagic machinery requires ubiquitination of the bacterial surface. This posttranslational modification process in which the small protein ubiquitin is covalently attached to bacterial target structures involves a cascade of enzymatic reactions mediated by E1 activating enzymes, E2 conjugating enzymes, and E3 ubiquitin ligases, with E3 ubiquitin ligases providing substrate specificity. When multiple ubiquitin moieties are linked, chains with the length of at least four ubiquitins are formed. These chains can be linked to different internal lysine residues or to the N-terminal methionine of ubiquitin, creating different chain structures that determine the functional outcome of the attached protein substrates influencing the cellular response (Komander and Rape, 2012).

During selective autophagy the ubiquitin chains attached to the pathogen can be recognized by autophagic adaptor proteins such as the ubiquitin-binding proteins p62/sequestosome-1 (SQSTM1), and nuclear dot protein 52 (NDP52). These proteins function as molecular bridges, thereby, linking ubiquitinated pathogens to the autophagosomal membrane through interactions with the microtubule-associated protein 1A/1B-light chain 3 (LC3) (Lamark et al., 2009; Ravenhill et al., 2019). LC3 plays a pivotal role in autophagy by facilitating the formation of autophagosomes through its lipidated form, LC3-II, which associates with autophagosomal membranes. Through further interactions with autophagy adaptor proteins, LC3 ensures the selective degradation of ubiquitinated pathogens, which is finally realized by the fusion of autophagosomes with cellular lysosomes (Sharma et al., 2018). The concept of bacterial surface ubiquitination was initially introduced in the context of *Salmonella* and *Listeria* infection, where it was identified as a cellular prerequisite for pathogen recognition and subsequent clearance (Perrin et al., 2004; Birmingham et al., 2006). In the last decade, similar ubiquitination processes have been described for *Mycobacterium tuberculosis* (Watson et al., 2012), *Francisella tularensis* (Chong et al., 2012), and *S. aureus* (Neumann et al., 2016). However, the knowledge about the E3 ubiquitin ligases involved in this process is still limited.

In this study, we investigated the function of the E3 ubiquitin ligase leucine rich repeat and sterile alpha motif containing 1 (LRSAM1), which has been previously implicated in the ubiquitination of the Gram-negative bacterium *Salmonella* Typhimurium (Huett et al., 2012). Our objective was to elucidate the impact of LRSAM1 on the fate of intracellular *S. aureus*. Our findings reveal a pivotal role for LRSAM1 in the elimination of intracellular bacteria, offering novel insights into the intricate mechanisms that govern intracellular bacterial clearance.

## 2 Materials and methods

### 2.1 Cell lines

The human alveolar epithelial cell line A549 (Lieber et al., 1976) was cultivated in RPMI1640 (Gibco) medium containing 10% fetal calf serum (FCS, Capricorn).

### 2.2 Bacterial strains

*S. aureus* HG001 strain (Herbert et al., 2010), a derivative of the NCTC 8325 strain with a repaired sigma B regulation factor (RsbU^+^) that restores full functionality of the stress sigma factor SigB, was maintained in tryptic soy broth (TSB, Roth) or on blood agar plates (BD Biosciences) at 37°C. The HG001-pMV158GFP (HG001-GFP) strain was generated as described before (Nieto and Espinosa, 2003) and plasmid maintenance was assured by addition of 20 µg/ml tetracycline to the culture medium. The HG001- pMV158GFPΔ*spa* strain (generated by S. Engelmann and J. Pané-Farré), used in order to prevent unspecific antibody staining in flow cytometry by binding of *S. aureus* Protein A to the Fc region of antibodies, was maintained by addition of 10 µg/ml erythromycin and 20 µg/ml tetracycline to the culture medium.

### 2.3 CRISPR-Cas9 genomic editing

CRISPR-Cas9 genomic editing for gene deletion was performed as previously described (Ran et al., 2013), using pSpCas9(BB)-2A-Puro (PX459) V2.0, a kind gift from Feng Zhang (Addgene plasmid #62988; http://n2t.net/addgene:62988; RRID: Addgene_62988). For *LRSAM1* gene deletion the sgRNA sequence 5´- CGTTCCATTGGGAACCTGAC-3´ targeting Exon 8 and for control cells non-targeting sgRNA 5´- ACATAGTCGACGGCTCGATT -3´ was cloned into the pSpCas9 (BB)-2A-Puro plasmid (pX459) and transfected into A549 cells. At 48 h transfected cells containing *LRSAM1*-specific and non-targeting sgRNA were placed under puromycin selection (5 µg/ml) for 2 days and afterwards diluted to single cells. Clones recovered post single cell dilution were picked, grown, and screened by immunoblot analysis. Additionally, genomic DNA was purified and amplified by PCR using the primers *LRSAM1*_for 5´- GCCGGTAGGGGGAGATAAGA-3´ and *LRSAM1*_rev 5´-GATGGAGGGTCACACGGAC-3´ designed to flank the site targeted by the sgRNA. The PCR products were validated by sequencing.

### 2.4 Infection of A549 cells

Infections were carried out as described before with minor adaptations (Palma Medina et al., 2019). In brief, prior infection of confluent epithelial cells, bacteria were grown in TSB medium up to an OD_600_ of 0.8 - 2. Bacteria were transferred to a pre-culture with a starting OD_600_ of 0.05 in RPMI1640 medium and grown to an OD_600_ of 0.3-0.5, to adapt the bacteria to the infection medium. The starting OD_600_ of the subsequent main culture was set to 0.05 and it was incubated for ∼2 h at 150 rpm and 37°C until it reached the mid-exponential phase at an OD_600_ of 0.4. We subsequently counted the cells from a separate well and calculated the volume of the bacterial culture to reach the desired MOI. The bacteria were then harvested accordingly and used for preparation of the master mix for infection. For preparation of fixed *S. aureus* HG001-GFP, the main culture was harvested at OD_600_ of 0.4 centrifuged and resuspended in PBS containing 2% paraformaldehyde (PFA, Applichem), incubated for 30 min at room temperature and subsequently washed with PBS to remove residual PFA and finally resuspended in RPMI. All infections were carried out at a multiplicity of infection of 10 (MOI 10) bacteria per host cell. The master mix for infection was prepared from a mid-exponential (OD_600_ of 0.4) culture of *S. aureus* HG001 diluted in RPMI, buffered with 2.9 μl sodium hydrogen carbonate (7.5%, PAN-Biotech GmbH) per ml bacterial culture added. A549 cells were seeded one day prior to infection in six-well plates. On the day of infection, the growth medium over the confluent epithelial layer was replaced with the infection master mix, and the co-culture was incubated for 1 h at 37°C in 5% CO_2_. Afterwards, the medium containing non-adherent bacteria was collected and replaced with fresh RPMI 1640 containing 10 µg/ml lysostaphin (Sigma) to follow up the internalization but avoiding reinfection. The infections were continued until the chosen time points and then processed for further analysis.

### 2.5 Colony forming unit determination

Cells were lysed at 0.5 h, 1 h, 2 h, 3 h, 5 h and 7 h post infection through hypotonic lysis using bidistilled water (*Aqua bidest*.) for 30 min. Serial dilutions from 10^-3^ – 10^-5^ of the released bacteria in *Aqua bidest.* were plated on blood agar plates. CFU counts were determined after 24 h incubation at 37°C and the mean CFU numbers were calculated from all dilutions. For normalization, a technical replicate of the infected cells was harvested using 1% trypsin (Capricorn Scientific) and subsequently counted using trypan blue staining to exclude dead cells in a counting chamber (Neubauer). Finally, CFUs were normalized to 1 x 10^5^ viable cells.

### 2.6 Fluorescence microscopy

Cells were seeded in 12-well cell culture plates containing autoclaved glass cover slips (Sarstedt). Infection with *S. aureus* HG001 (MOI 10) was carried out as described above (2.1.4.). At 3 h post infection cells were washed with PBS and subsequently fixed with 2% paraformaldehyde in PBS for 10 min. After fixation, the cells were washed twice with PBS and then permeabilized with 1 mL per well of 0.1% Triton X-100 in phosphate-buffered saline (PBS, Th. Geyer) for 5 minutes at room temperature. After subsequent washing with PBS cells were stained as described before (Hildebrandt et al., 2016) with the staining solution containing 1 µg/ml HOECHST 33258 (Sigma-Aldrich), 3 U/ml Flash Phalloidin-red 594 (Biolegend) and 0.4 µg/µl Vancomycin, BODIPY™ FL Conjugate (Thermo Fisher Scientific) and incubated for 10 min at room temperature in the dark. After staining the cells were washed twice with PBS and once with *Aqua bidest.* The cover slips were transferred to an object slide with Fluorescent Mounting Medium (DAKO). Pictures were acquired for each cover slip using a REVOLVE microscope (Discover Echo) and processed with the included Echo Pro software.

### 2.7 Flow cytometry

Cells were harvested using 1% trypsin (Capricorn Scientific) at indicated time-points post infection. Cells infected with *S. aureus* HG001-pMV158GFP were washed once with PBS and directly measured via flow cytometry. For ubiquitin staining of *S. aureus* HG001-pMV158GFP Δ*spa*, infected cells were treated with *Aqua bidest*. for 30 min, and subsequently centrifuged at 10000 g for 2 min, washed once with PBS and stained with anti-ubiquitin (P4D1)-Alexa 647 antibody (Santa Cruz) for 20 min in the dark. For ubiquitin chain-specific staining cells were stained with primary antibodies for K48- and K63-ubiquitin chains (all Cell Signaling Technology) for 30min and after subsequent washing stained with anti-rabbit-Alexa-647 secondary antibody (Abcam) for 30min. The background staining of the secondary antibody was evaluated using the secondary antibody alone as a control, with no primary antibodies present. Data were acquired using a MACSQuant Analyzer 10 Flow Cytometer (Miltenyi Biotec) and analyzed with FlowJo™ Version 10.6.0 software. For assessing the bacteria, the FSC and SSC triggers were set to 0, due to the small size of the bacteria.

### 2.8 Immunoblot analysis

Proteins were isolated using TRIzol^©^ reagent (Thermo Fisher Scientific) according to manufacturer´s instructions and quantified via Bradford assay. For immunoblotting proteins were separated by SDS-PAGE and transferred to nitrocellulose membranes. After subsequent blocking with ROTI^®^Block (Carl Roth GmbH) membranes were analyzed for target proteins using primary antibodies for PARP, cleaved PARP, LRSAM1, pIκBα, IκBα, LC3, pMLKL, MLKL and β-actin (all Cell Signaling Technology). After incubation with primary antibodies membranes were incubated for 60 min at RT with species-specific and horseradish peroxidase-coupled secondary antibodies. Membranes were developed with chemiluminescence using SignalFire™ ECL Reagent (Cell Signaling Technology) and analyzed with Imagequant 800 system (Cytiva).

### 2.9 Cell viability assays

The monitoring of cell viability was performed by analyzing the LDH content in cell supernatants by CytoTox-ONE Homogeneous Membrane Integrity Assay (Promega) according to manufacturer’s protocol. In brief, 50 µL of supernatants were incubated for 10 min with 50 µL substrate solution. Afterwards the reaction was stopped by adding 25 µL of Stop solution. Supernatants of non-infected cells treated for 10 min at 37°C with 10% TritonX 100 (AppliChem) served as positive control reflecting 100% cell death. Samples were measured at 590 nm using a plate reader (Tecan).

### 2.10 Cytokine analysis

ELISA for IL-6 (BioLegend) was performed according to manufacturer’s instructions. In brief, plates were coated with the included capture antibody and incubated for 16-18 h at 4°C. Plates were washed (PBS + 0.5% Tween-20). Hereafter, plates were blocked with blocking solution for 1 h with shaking at 500 rpm. Furthermore, standard series were prepared (IL-6, 500 pg/ml – 7.8 pg/ml). Non-specific bindings were blocked, blocking solution was removed and plates were washed. Standards and cell culture medium samples were added to the plates and incubated for 2 h with shaking. After the plates were washed, they were incubated with the biotinylated detection antibody for 1 h. The detection antibody solution was removed, and plates were washed again. Avidin-HRP solution was added and incubated for 30 min with shaking. Avidin-HRP solution was removed, and plates were washed again. TMB substrate solution was added and incubated for 15 min in the dark. Stop solution (2 N H_2_SO_4_) was added and absorbance was measured at 450 nm within 15 min.

### 2.11 Cathepsin B measurement

To assess cellular cathepsin B activity cells were lysed post infection using 10 mM Tris-HCl (pH 7.0), 10 mM NaCl, 25 mM KCl, 1 mM MgCl_2_, 10% Glycerol and 1mM Dithiothreitol (DTT) with three repeated freeze-thaw cycles of 15s incubation in liquid nitrogen and 3 min at 37°C. Protein concentration was determined via Bradford assay. The enzyme reaction was started by adding the substrate R-R-AMC (Bachem) to 2 µg protein lysate in assay buffer containing 100 mM Na-acetate (pH 5.5), 5 mM CaCl_2_ and 10 mM DTT as described before (Sendler et al., 2016). Enzyme kinetics were measured 0 – 1 h using a fluorescence plate reader (Tecan) with an excitation wavelength of 360 nm and an emission wavelength of 460 nm. Calculated values are presented as change in relative fluorescence per minute compared to baseline.

## 3 Results

**Figure 1:**
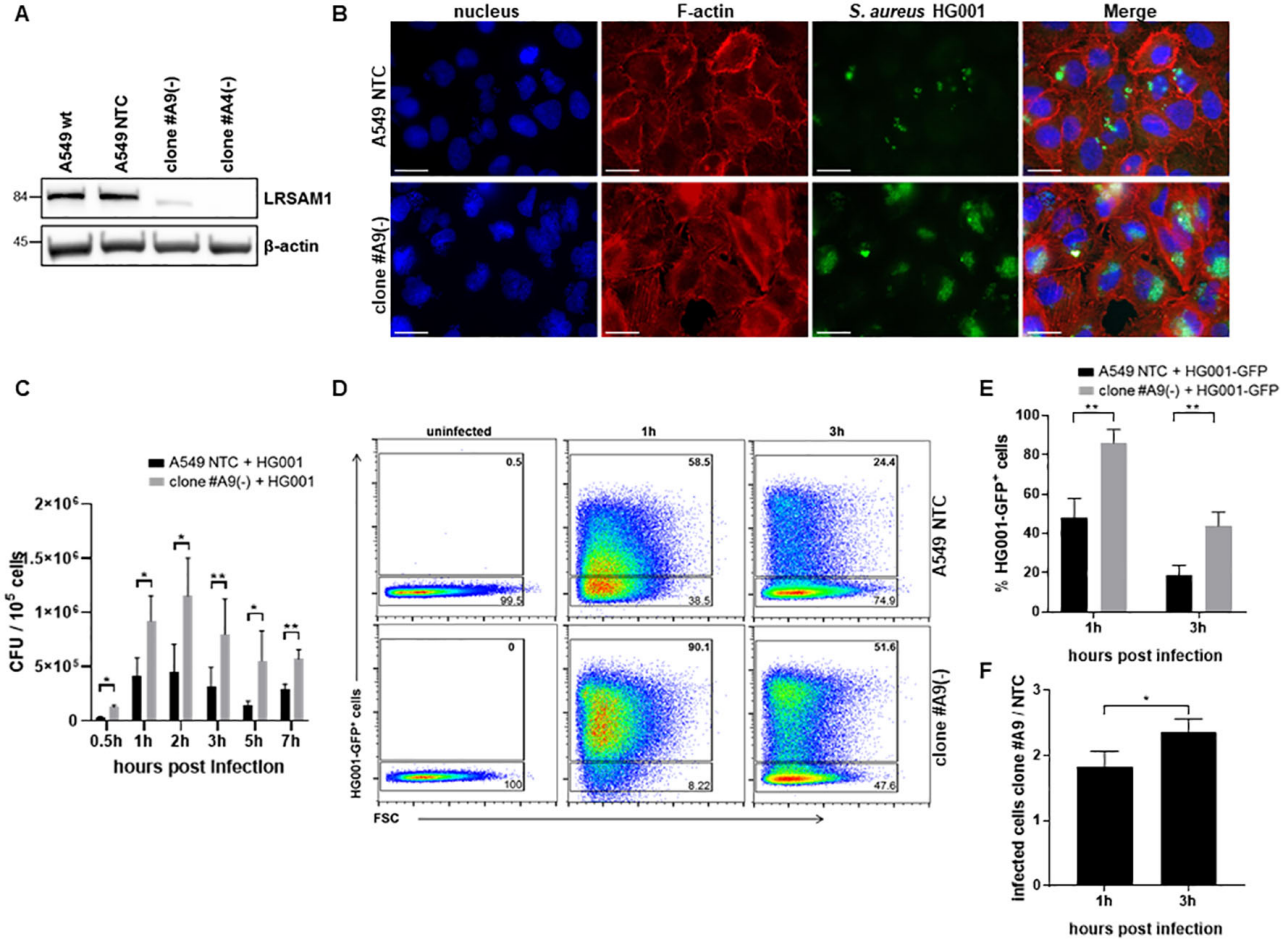
Higher intracellular bacterial load upon deletion of LRSAM1 in A549 epithelial cells. **(A)** CRISPR CAS9 mediated deletion of LRSAM1 was confirmed by immunoblot for clone #A9 and #A4 and compared to control cells (A549 wt and A549 NTC). **(B)** LRSAM1 KO (clone #A9) and control cells (A549 NTC) were infected with *S. aureus* HG001 (MOI 10) and subsequently stained with HOECHST 33258 – nucleus, Flash Phalloidin-red 594 – F-actin and Vancomycin, BODIPY™ FL Conjugate - bacteria for fluorescence microscopy 3 h post infection, n = 3, magnification 60x, scalebar 20 µm **(C)** LRSAM1 KO (clone #A9) and control cells (A549 NTC) were infected with *S. aureus* HG001 (MOI 10), cells were lysed at the depicted time-points and the released bacteria were plated on blood agar plates for determination of the colony forming units (CFU), data is represented as mean ± SD of three replicates. Statistical significance is indicated (*p<0.05, **p<0.01, students t-test) **(D)** LRSAM1 KO (clone #A9) and control cells (A549 NTC) were infected with *S. aureus* HG001-GFP (MOI 10) and analyzed for the number of infected cells by flow cytometry at the depicted time points. **(E)** Data analysis is represented as mean ± SD of three replicates. Statistical significance is indicated (**p<0.01, students t-test). **(F)** The ratio between infected LRSAM1-deficient and infected NTC cells is depicted for 1 h and 3 h upon infection. Ratios are presented as mean ± SD of three replicates. Statistical significance is indicated (*p<0.05, students t-test).

The objective of this study was to assess the role of LRSAM1 in the recognition and clearance of intracellular *S. aureus* HG001 by infected human alveolar epithelial cells. To this end, a CRISPR-CAS9-mediated deletion for LRSAM1 was generated in A549 lung epithelial cells (clones #A4 and #A9, **Figure 1A** and **Supplementary Figure 1A**). Given that A549 epithelial cells are non-professional phagocytic cells, we hypothesized that the deletion of LRSAM1 would have an effect on the uptake and amount of intracellular bacteria. To test this, wild-type (non-targeted control, NTC) and LRSAM1-deficient cells (clone #A9) were infected with *S. aureus* HG001, and analyzed for the bacterial intracellular life style as done before (Surmann et al., 2015). Intracellular bacteria were stained with fluorescence-labelled vancomycin, BODIPY™ FL, and were monitored via fluorescence microscopy. The results demonstrated an increased intracellular bacterial load in LRSAM-deficient cells at 3 h post infection (**Figure 1B**; **Supplementary Figure 1B**).

To address the question whether the intracellular bacteria are still viable, we lysed the host cells and plated the non-stained bacteria on blood agar plates to determine the colony forming units (CFUs). Infection of both LRSAM1 KO and NTC cells showed a marked increase in viable intracellular bacteria at 0.5 h, 1 h and 2 h, followed by decreased bacterial loads at 3 h and 5 h. Throughout the infection, LRSAM1 KO clones #A9 and #A4 consistently exhibited higher intracellular CFU counts compared to control cells (**Figure 1C**; **Supplementary Figure 1C**).

Next, we investigated whether the increased bacterial load observed in LRSAM1 knockout cells was attributable to enhanced invasion and/or improved intracellular survival of *S. aureus*. Since we removed all extracellular bacteria 1 hour after infection by adding lysostaphin, *S. aureus* invasion can only occur during the first hour of infection. Within this timeframe, we observed a significant rise in intracellular bacteria, coupled with a slight decrease in CFUs of extracellular bacteria in the infection medium of the LRSAM KO cells compared to NTC cells (**Figure 1C**; **Supplementary Figure 1D**). At later time points investigating *S. aureus* intracellular survival by infecting the cells with the HG001-GFP strain, we demonstrated that a higher percentage of host cells was infected 1 h post-infection than 3 h post-infection, with a significantly higher rate of infected cells in LRSAM1-deficient than in NTC cells (**Figures 1D, E**). In addition, an increase in the ratio of infected LRSAM1-deficient to NTC cells was observed from 1 h to 3 h post infection, indicating reduced clearance of intracellular *S. aureus* in the LRSAM1-deficient cells (**Figure 1F**). These results suggest that LRSAM1-deficient cells have an increased susceptibility to *S. aureus* invasion and an inability to clear intracellular bacteria compared to control cells. To address the question of whether *S. aureus* must be viable to exhibit this phenotype, we infected cells with PFA-fixed *S. aureus* HG001-GFP. We observed only a very low number of intracellular bacteria, with no difference between the LRSAM1 KO and NTC (**Supplementary Figure 1E**).

**Figure 2:**
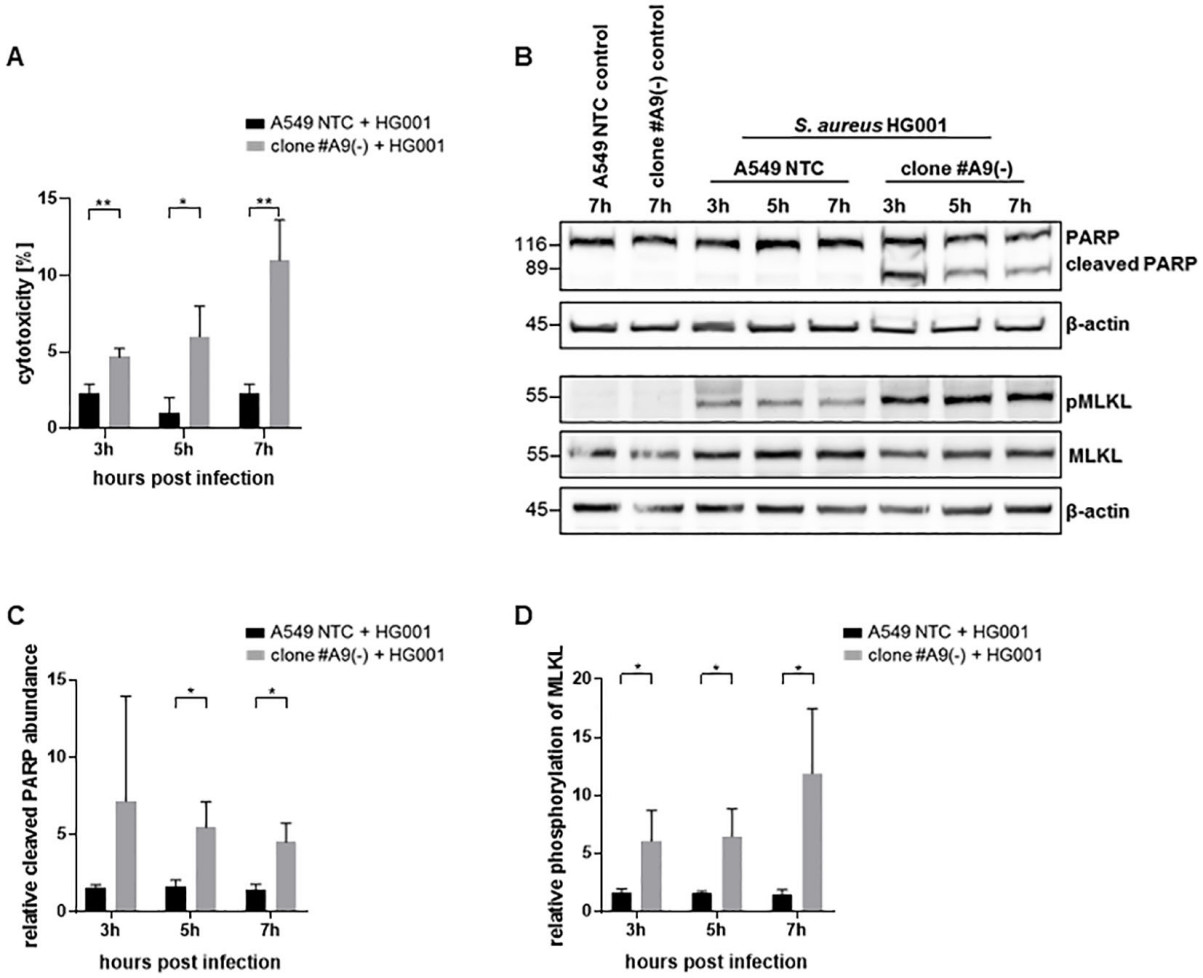
Increased cell death in infected LRSAM1-deficient cells. **(A)** Host cell death was monitored upon infection with *S. aureus* HG001 (MOI 10) by determining extracellular lactate dehydrogenase (LDH) at the depicted time-points comparing LRSAM1 KO (clone #A9) and control cells (A549 NTC). Data are represented as mean ± SD of three replicates. Statistical significance is indicated (*p<0.05, **p<0.01, students t-test) **(B)** LRSAM1 KO (clone #A9) and control cells (A549 NTC) were infected with *S. aureus* HG001 (MOI 10) for the depicted time-points and subsequently analyzed for PARP cleavage and phosphorylation of MLKL and total MLKL by immunoblotting with β-actin as loading control. Band intensities were analyzed by densitometry. Cleaved PARP was normalized to the loading control β-actin and phosphorylated MLKL was normalized to MLKL and the loading control β-actin. Graph depicts relative cleaved PARP **(C)** and relative phosphorylation of MLKL **(D)** in LRSAM1 KO (clone #A9) and control cells (A549 NTC) calculated to the respective non-infected control. Data are represented as mean ± SD of three replicates. Statistical significance is indicated (*p<0.05, students t-test).

To determine whether the elevated bacterial load and infection rate impact host cellular survival, we investigated cell death induction during infection. By assessing extracellular LDH release, we observed an elevated cell death 3 h, 5 h, and 7 h post infection in LRSAM1-deficient cells compared to NTC cells (**Figure 2A**; **Supplementary Figure 2A**). It is known from previous studies that *S. aureus* infection can induce apoptosis and necroptosis in the host cells (Haslinger-Löffler et al., 2005; Greenlee-Wacker et al., 2017). To further refine the observed increased cell death, we analyzed the cleavage of the apoptotic marker poly(ADP-ribose)-polymerase (PARP) and the phosphorylation of mixed lineage kinase domain like pseudokinase (MLKL), which is a hallmark in necroptosis. Levels of both markers were significantly increased in infected LRSAM1 knockout cells compared to NTC cells. These results indicate *S. aureus*-specific induction of host cell death in LRSAM1-deficient cells (**Figures 2B-D**). Furthermore, infection with PFA-fixed bacteria had no impact on the induction of cell death in either LRSAM1-deficient cells or NTCs (**Supplementary Figures 2B, C**). This suggests that active expression of virulence factors is necessary for this process.

**Figure 3:**
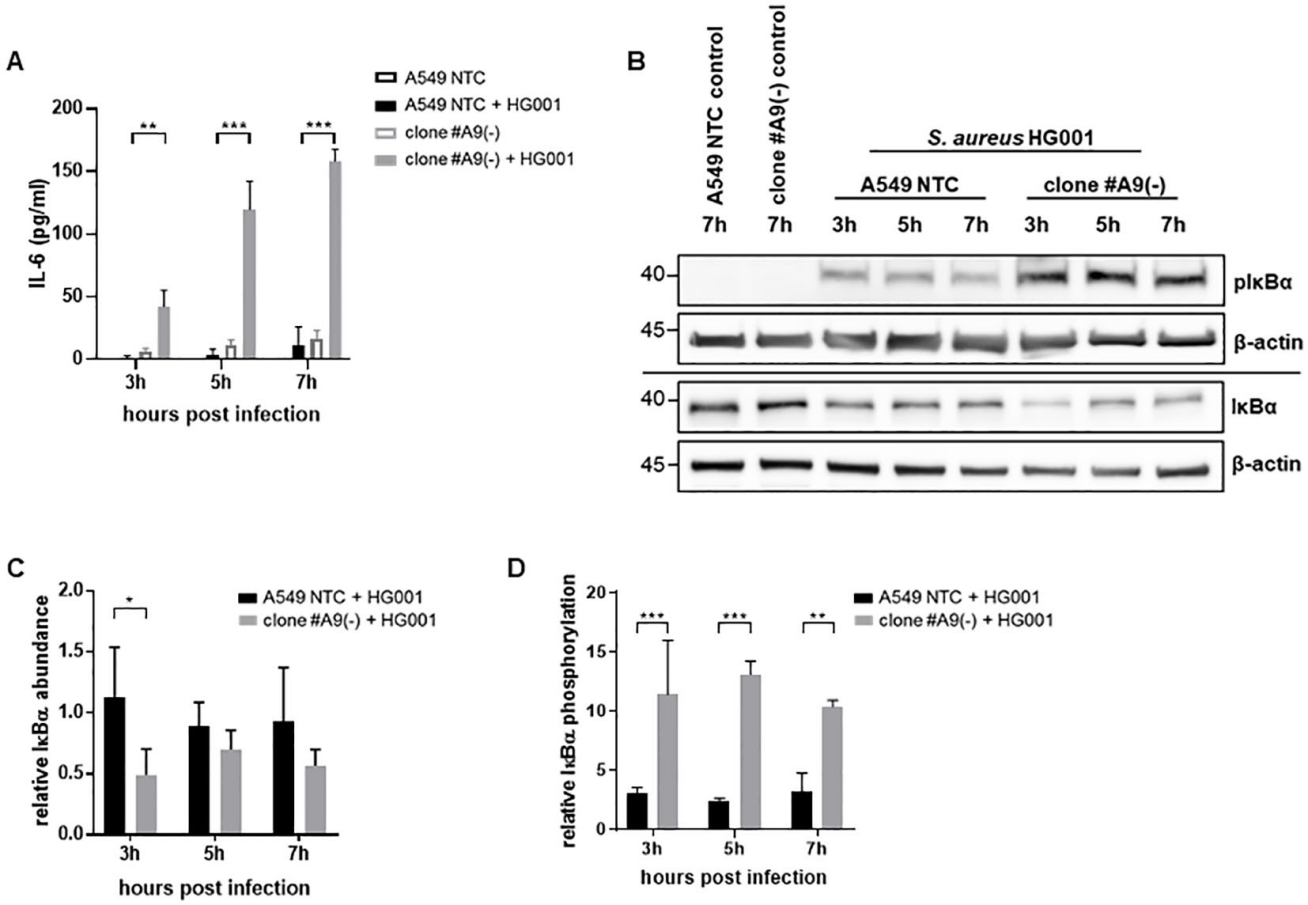
Elevated levels of secreted IL-6 in LRSAM1-deficient cells during infection. **(A)** Secretion of IL-6 was determined by ELISA for the depicted time points in cell culture supernatants of LRSAM1 KO (clone #A9) and control cells (A549 NTC) during infection with *S. aureus* HG001 (MOI 10). Data are represented as mean ± SD of three replicates. Statistical significance is indicated (**p<0.01, ***p<0.001, students t-test) **(B)** LRSAM1 KO (clone #A9) and control cells (A549 NTC) were infected with *S. aureus* HG001 (MOI 10) for the depicted time-points and subsequently analyzed for pIκBα and IκBα by immunoblotting with β-actin as loading control. **(C)** Band intensities of IκBα were analyzed by densitometry and normalized to the loading control β-actin. Graph depicts relative IκBα in LRSAM1 KO (clone #A9) and control cells (A549 NTC) calculated to the respective non-infected control, Data are represented as mean ± SD of three replicates. **(D)** Band intensities of pIκBα were analyzed by densitometry and normalized to the loading control β-actin. Graph depicts relative pIκBα in LRSAM1 KO (clone #A9) and control cells (A549 NTC) calculated to the respective non-infected control, Data are represented as mean ± SD of three replicates. Statistical significance is indicated (*p<0.05, **p<0.01, ***p<0.001, students t-test).

*S. aureus* virulence factors such as protein A or α-toxin can trigger MAPkinase pathways, which in turn lead to the activation of transcription factors such as NFκB (Gómez et al., 2004; Räth et al., 2013). This activation is followed by an induction and secretion of pro-inflammatory cytokines like IL-6, which mediates the recruitment of innate immune cells like neutrophils and macrophages to the site of infection (Ngo et al., 2022). During infection of LRSAM1-deficient cells, a significant increase in IL-6 secretion was observed compared to NTC cells (**Figure 3A**; **Supplementary Figure 2D**). Since the secretion of IL-6 is dependent on the activation of the NFκB-pathway, we addressed the question if LRSAM1-deficiency impacts NFκB induction by assessing the expression level of the NFκB-inhibitory protein IκBα and its phosphorylation. The results demonstrated an increase in IκBα degradation as well as an increased phosphorylation of IκBα during infection in LRSAM1-deficient cells (**Figures 3B–D**), indicating an elevated activity of the NFκB-pathway with increased secretion of IL-6 upon infection.

**Figure 4:**
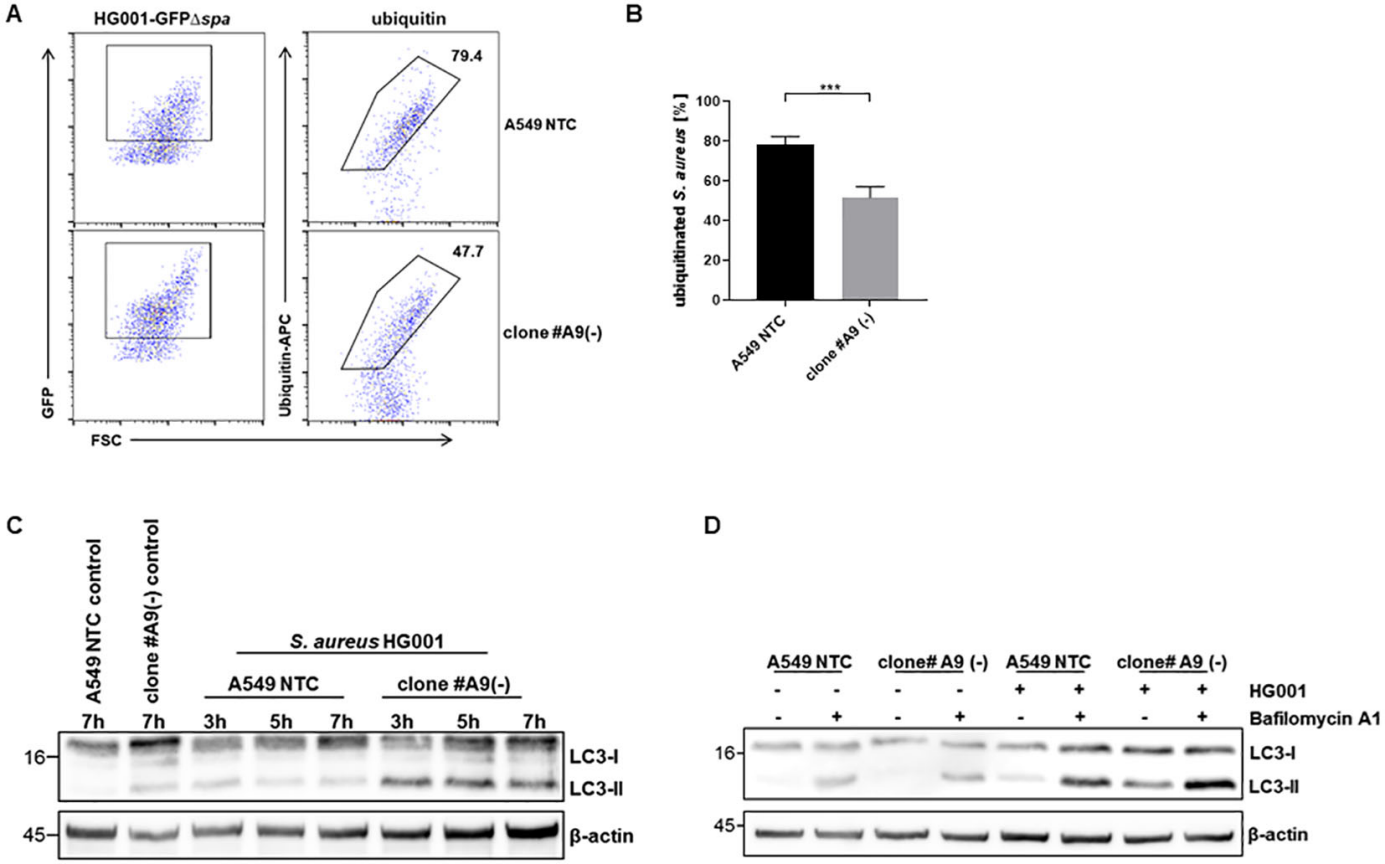
LRSAM1-deficient cells show induced cellular autophagy in response to *S. aureus* infection. **(A)** LRSAM1 KO (clone #A9) and control cells (A549 NTC) were infected with *S. aureus* HG001-GFPΔ*spa* (MOI 10), which was used to prevent unspecific binding of the anti-ubiquitin antibody to *S. aureus* Protein A, and lysed 3 h post infection. Released intracellular bacteria were analyzed for ubiquitination by staining with anti-APC-ubiquitin antibody and subsequent flow cytometry analysis by gating on 1000 GFP-positive bacteria in total. **(B)** Graph depicts the percentage of ubiquitinated intracellular *S. aureus*, n = 4, statistical significance is indicated (***p<0.001, students t-test). **(C)** LRSAM1 KO (clone #A9) and control cells (A549 NTC) were infected with *S. aureus* HG001 (MOI 10) for the depicted time-points and subsequently analyzed for LC3-I and LC3-II by immunoblotting with β-actin as loading control. One representative blot out of four replicates is shown. **(D)** LRSAM1 KO (clone #A9) and control cells (A549 NTC) were treated with 100 ng/mL Bafilomycin A1 1 h prior infection or left untreated and infected with *S. aureus* HG001 (MOI 10) for 3 h and subsequently analyzed for LC3-I and LC3-II expression by immunoblotting with β-actin as loading control, n = 3.

Given the observation of an accumulation of intracellular *S. aureus* in LRSAM1-deficient cells, we asked whether ubiquitination and selective autophagy were impaired in these cells. Ubiquitination of the bacterial surface is a crucial prerequisite for the recognition and subsequent elimination of intracellular bacteria. To test this, intracellular bacteria were isolated from infected host cells, stained with an anti-ubiquitin antibody and analyzed via flow cytometry. For this analysis, we used the *S. aureus*-GFPΔ*spa* strain to prevent non-specific binding of the antibodies to Protein A. This strain exhibited a comparable intracellular bacterial phenotype to that of the wild-type *S. aureus* HG001 strain (**Supplementary Figure 3A**; **Figure 1C**). The results indicated that bacterial ubiquitination was significantly reduced in LRSAM1 KO cells (**Figures 4A, B**; **Supplementary Figure 3B**). Moreover, we could observe the formation of both K48 and K63 ubiquitin chains at the bacterial surface, which was reduced in the LRSAM1 KO cells to a similar extent (**Supplementary Figure 3C**).To analyze whether the reduced ubiquitination has an impact on subsequent autophagy induction we examined the conversion of the autophagy marker LC3-I to LC3-II by immunoblotting. We observed an enhanced LC3-II formation in infected LRSAM1-deficient cells compared to NTC cells (**Figure 4C**). These observations suggest that the elevated intracellular bacterial load increased autophagic activity. However, due to reduced ubiquitination, the bacteria could not efficiently be removed from the cells through autophagy. To validate whether the increase in LC3-II resulted from an enhanced autophagic flux or a block in autophagy downstream, we added the lysosomal inhibitor bafilomycin A1 during the infection. A substantial increase in LC3-II in the bafilomycin-treated samples is evidence against a block of autophagy, indicating an elevated autophagic turnover in the infected cells (**Figures 4D**). Furthermore, we measured the activity of cathepsin B, a lysosomal protease necessary for intracellular proteolysis and elimination of bacteria in (auto-)phagosomal vesicles. We observed an increase in cellular cathepsin B activity in LRSAM1-deficient cells upon infection, which confirmed the higher activity of autophagy in these cells compared to NTC control cells (**Supplementary Figure 3D**).

## 4 Discussion

*S. aureus* infections continue to pose substantial therapeutic challenges, underscoring the necessity for novel treatment strategies. A critical concern in clinical settings is the ability of *S. aureus* to persist and survive within host cells, a capability that renders conventional antibiotic treatments ineffective (Rodrigues Lopes et al., 2022). Consequently, there is an imminent need to enhance our understanding of the intricate interactions between *S. aureus* and its host cells. The ability of *S. aureus* to persist intracellularly varies depending on the type of host cell. Epithelial cells are invaded by *S. aureus* and can serve as a reservoir for chronic or recurrent infections. This applies to keratinocytes in skin infections or epithelial cells of the respiratory tract causing pneumonia (Morgenstern Alexis et al., 2024; Lacoma et al., 2017; Stelzner et al., 2021). Epithelial cells represent the first line of defense against invading pathogens. While these cells do not actively phagocytose, studies have demonstrated the ability of *S. aureus* to invade, survive, and persist within these cells especially by formation of the SCV phenotype (Tuchscherr et al., 2010, 2011; Proctor et al., 2014; Kahl et al., 2016). However, host cells have evolved mechanisms to combat and eliminate bacteria. A more profound understanding of these processes will facilitate the development of novel strategies for treating *S. aureus* infections.

In this study, we examined the function of the ubiquitin E3 ligase LRSAM1 in the recognition and elimination of intracellular *S. aureus* HG001 in A549 alveolar epithelial cells. Consequently, HG001 has been employed to study intracellular host-pathogen interactions, as it is capable of surviving and replicating within the intracellular environment (Mauthe et al., 2012; Surmann et al., 2014; Palma Medina et al., 2019). Our experimental infection model, which utilized the infection of A549 NTC cells as controls, corroborated these findings, displaying intracellular bacterial survival, low levels of cell death, and low secretion of the cytokine IL-6.

Upon deletion of LRSAM1 this phenotype dramatically changed. We observed a higher infection rate and larger numbers of intracellular bacteria indicating that LRSAM1 is critical for controlling intracellular *S. aureus*. Comparable results have been obtained in a study analyzing *Salmonella* infections in HeLa cells where downregulation of LRSAM1 via siRNA increased the intracellular numbers of *Salmonella* significantly (Huett et al., 2012). Moreover, we could demonstrate that the invasion of *S. aureus* in general depends on bacterial viability. Besides the increased bacterial load, we observed a higher infection rate, which might indicate an increased susceptibility of the host cells to bacterial invasion (**Figure 1D**). A reason for this might be LRSAM1’s ubiquitin ligase activity which has been shown to influence receptor sorting during endocytosis by regulating key endosomal sorting proteins, particularly tumor susceptibility gene 101 (TSG101), a core component of the endosomal sorting complex required for transport-I (ESCRT-I) complex, which is essential for the endosomal sorting and lysosomal degradation of receptors such as the epidermal growth factor receptor (Mishra et al., 2019). In contrast, studies of *Salmonella* infection showed that knockdown of the autophagy receptors NDP52 and p62, which are responsible for recognizing ubiquitinated *Salmonella*, had no effect on the susceptibility of cells to the bacteria (Thurston et al., 2009; Zheng et al., 2009).

Many studies documented the ability of *S. aureus* to induce host cell death, a process frequently characterized by the activation of programmed cell death pathways, including apoptosis, necroptosis, and inflammasome-mediated pyroptosis (Esen et al., 2001; Haslinger-Löffler et al., 2005; Greenlee-Wacker et al., 2017; Melehani et al., 2015). In accordance with these findings, our results revealed an increase in apoptotic and necroptotic cell death during infection, which was more pronounced in LRSAM1-deficient cells. It is important to note that other studies analyzing host cell death mechanisms upon *S. aureus* infection applied higher MOIs (≥50) than used in our experimental set up. Our findings suggest the following scenario: LRSAM1 deletion results in elevated intracellular bacterial levels, which lead to higher induction of apoptosis and increased necroptosis compared to NTC control cells. However, no pyroptotic cell death was observed (data not shown). In addition, the induction of cell death depends on the active expression of virulence factors by viable *S. aureus*. Therefore, PFA-fixed *S. aureus* had no impact on these processes in A549 cells. The same has also been shown for HUVEC cells, for which apoptosis induction requires staphylococcal invasion and metabolically active intracellular staphylococci (Haslinger-Löffler et al., 2005). Moreover, a high intracellular bacterial load in the LRSAM1-deficient cells could also explain the increased activation of the NFκB pathway, which subsequently leads to the profound IL-6 secretion observed. Similar results were shown for keratinocytes infected with *S. aureus* 8325-4, where a clear relationship between the amount of intracellular *S. aureus* and IL-6 production was demonstrated (Ngo et al., 2022). However, since the uninfected LRSAM KO cells also showed a slight increase in IL6 secretion, it is possible that LRSAM1 KO cells are more sensitive to extracellular stimuli for cytokine secretion. One possible reason for this is the proposed role of LRSAM1 in receptor endocytosis. The ubiquitination of the endosomal sorting protein TSG101 has been shown to be essential for the lysosomal degradation of receptors (Mishra et al., 2019). A LRSAM1 knockout may stabilize surface receptors, thereby sensitizing the cells to extracellular stimuli, which may manifest at later time points due to e.g., cells’ high confluency.

Ubiquitination of bacteria is crucial for their elimination via selective autophagy (Goodall et al., 2022). Assessing surface ubiquitination of *S. aureus* revealed a significant reduction; however, ubiquitination was not abrogated in LRSAM1-deficient cells. This suggests that other E3 ligases may partially compensate for the loss of LRSAM1. The reduced ubiquitination in LRSAM1-deficiency may explain the observed increase in intracellular bacteria. To date, few E3 ligases are known to ubiquitinate intracellular bacteria. RNF213 is implicated in recognizing lipopolysaccharide (LPS) and ubiquitinating *Salmonella* (Otten et al., 2021). PARKIN and SMURF1 have been associated with *M. tuberculosis* ubiquitination (Manzanillo et al., 2013; Franco et al., 2017). Additionally, ARIH1 has been observed on *Salmonella* surfaces prior to LRSAM1 during infection (Polajnar et al., 2017). This highlights the complexity of the host’s ubiquitination machinery in response to intracellular pathogens. Variations in the type of ubiquitin chain catalyzed by different E3 ligases may further affect the efficiency with which *S. aureus* is targeted by selective autophagy. We observed both K48- and K63-linked ubiquitin chains at the surface of *S. aureus*. Since both chain-types were reduced in the LRSAM1 KO, LRSAM1 may be responsible for the generation of these chains. Although LRSAM1 has been shown to be capable of creating all ubiquitin linkage types except M1, it is known to preferentially create K6 and K27 ubiquitin chains (Huett et al., 2012) whereas other E3 ligases such as PARKIN and SMURF1 facilitate K63 and K48 ubiquitin chains, respectively, on intracellular *M. tuberculosis* (Manzanillo et al., 2013; Franco et al., 2017). As LRSAM1 is not the only E3 ligase responsible for ubiquitinating *S. aureus*, future studies must address which observed linkage types might be attributed to LRSAM1 or alternative E3 ligases, which may act alone or together with LRSAM1 in a “bacteria-recognition-complex”. In spite of the reduced ubiquitination, we observed a robust induction of autophagy, as evidenced by elevated levels of LC3-II. Given that LC3-II is degraded along with its bound cargo within the autophagosomes, elevated levels of LC3-II may indicate either a blocked autophagic pathway or an augmented autophagic flux. Treatment with bafilomycin, which impedes the fusion of the autophagosomes with lysosomes, resulted in accumulation of LC3-II, suggesting an increased autophagic flux. The overall role of autophagy in *S. aureus* infection remains controversial in the literature. In addition to its proposed role in eliminating intracellular bacteria, there is evidence that autophagosomes may serve as intracellular niches that are essential for *S. aureus* replication (Schnaith et al., 2007). The marked induction of autophagy in LRSAM1-deficient cells during *S. aureus* infection suggests a potential exploitation of autophagy for replication by *S. aureus*. On the other hand, it has been proposed that *S. aureus* can actively evade autophagy-mediated degradation by manipulating the host MAPkinase p38 during the infection process resulting in bacterial replication in the cytosol (Neumann et al., 2016). Moreover, it has been shown that *S. aureus* can induce autophagy without being targeted to autophagosomal degradation, by modifying the host cell metabolism, thereby, inducing starvation-induced autophagy, resulting in the production of *S. aureus*-free autophagosomes within HeLa cells (Bravo-Santano et al., 2018). In addition, pattern recognition receptors (PRRs), notably Toll-like receptor 2, sense bacterial components and influence autophagy signaling pathways (Wang et al., 2022), thereby inducing autophagy in response to infection. The varied outcomes observed in the host cellular responses to intracellular *S. aureus* may be indicative of the diverse strain-specific differences in intracellular survival, which are often attributable to differential expression of bacterial virulence factors. Additionally, the type of host cell infected makes a great difference as well (Grosz et al., 2014; Bayer et al., 2024).

**Figure 5:**
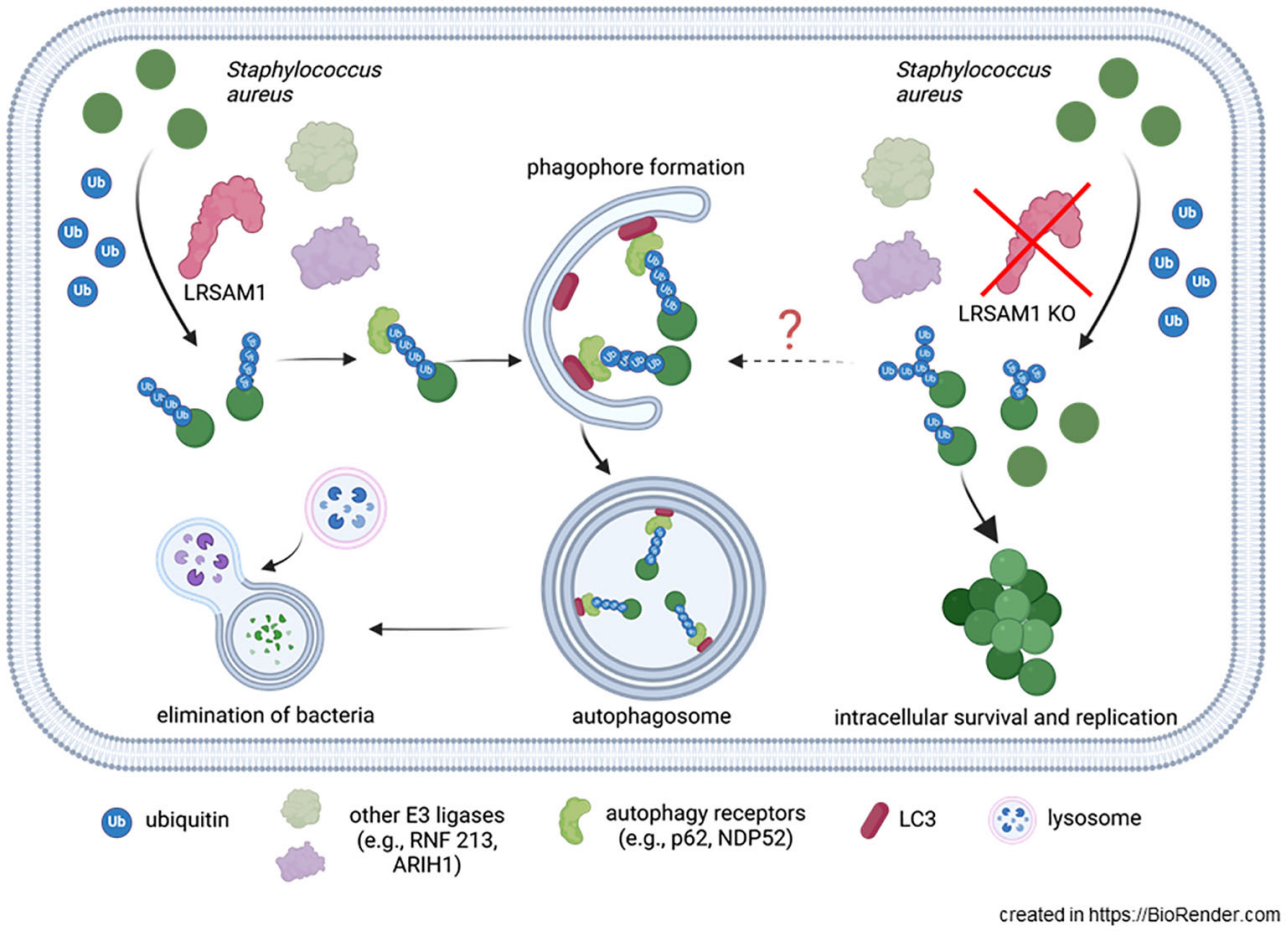
Model of ubiquitination and subsequent elimination of *S. aureus* via LRSAM1. *S. aureus* is ubiquitinated by LRSAM1 within the cytosol. The ubiquitinated bacteria are recognized by autophagy adaptor proteins, including NDP52 and p62, which facilitate interaction with LC3, resulting in phagosome maturation and subsequent fusion with lysosomes, leading to bacterial elimination. In the absence of LRSAM1, alternative E3 ligases can compensate the loss of ubiquitination by LRSAM1 ubiquitinating *S. aureus* albeit to a lesser extent or attaching different ubiquitin chains. This, in turn, reduces the recognition of *S. aureus* by the autophagic adaptor proteins, thereby facilitating its intracellular survival and replication.

Taken together, our data show a pivotal role for LRSAM1 in *S. aureus* HG001 ubiquitination and elimination via selective autophagy. Our current simplified model, highlighted in **Figure 5**, suggests that LRSAM1 ubiquitinates intracellular *S. aureus* in the cytoplasm upon infection. The ubiquitinated bacteria are recognized by autophagic adaptor proteins such as NDP52 and p62, which mediate interaction with LC3, leading to phagosome maturation and ultimately lysosome fusion, which eliminates the bacteria. In LRSAM1-deficient cells, this pathway is shut down. However, alternative E3 ligases can compensate for the loss of LRSAM1, resulting in partial ubiquitination and potential differences in the type of ubiquitination and ubiquitin chain linkages that compromises subsequent autophagy. Therefore, the host cell has a reduced capacity to eliminate *S. aureus*, leading to uncontrolled intracellular replication, a “paradoxical” increase in total autophagic activity and induction of programmed host cell death. Several questions remain to be addressed in future research. For example, is there a chronological sequence of different E3 ligases whose activity builds on each other, and what are the actual targets that are ubiquitinated on the bacterial surface?

Our findings contribute to the understanding of how *S. aureus* is targeted by the ubiquitination machinery of the host, particularly through LRSAM1, and how this modification influences selective autophagy and subsequent bacterial elimination. The interplay between bacterial evasion strategies and host defense mechanisms underscores the dynamic nature of host-pathogen interactions during intracellular *S. aureus* infection. By elucidating these intricate mechanisms, we establish a foundation for the development of alternative approaches such as the development of bacteria specific Proteolysis Targeting Chimeras (PROTACs) to force the intracellular elimination of *S. aureus* in patients.

## Data Availability

The raw data supporting the conclusions of this article will be made available by the authors, without undue reservation.
