# Supplementary material for: Deletion of the E3 ubiquitin ligase LRSAM1 fosters intracellular Staphylococcus aureus survival
Source: Front Cell Infect Microbiol. 2025 Aug 11;15:1597830. doi: 10.3389/fcimb.2025.1597830 (PMC12375608; doi:10.3389/fcimb.2025.1597830)
Supplement: Supplementary file 1 [file Supplementaryfile1.docx]

Supplementary Material


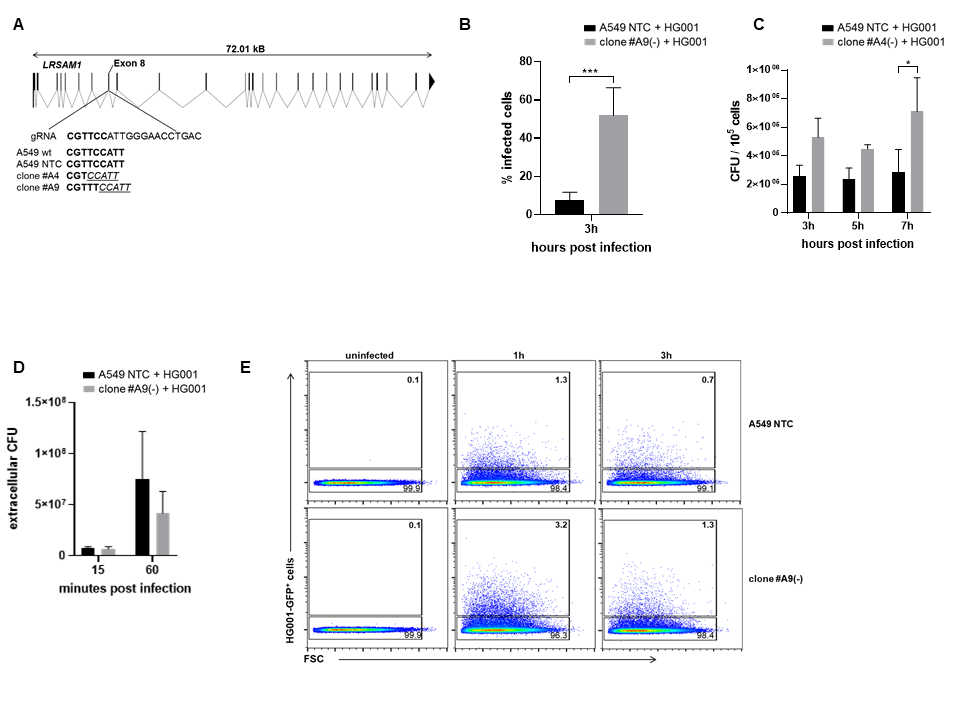


**Supplementary Figure 1.**

**(A)** Sequencing of the genomic DNA of clones #A4 and #A9 confirmed CAS9-mediated cleavage at the designated site in exon 8 introduced by the gRNA sequence compared to the A549 parental cell line. Wild type sequences for A549 parental cell line and CRISPR Cas9 control cells (A549 NTC) are printed in bold, missense sequences after CAS9 mediated cleavage are underlined in italics. **(B)** LRSAM1 KO (clone #A9) and control cells (A549 NTC) were infected with *S. aureus* HG001 (MOI 10) and subsequently stained with HOECHST 33258 – nucleus, Flash Phalloidin-red 594 – F-actin and Vancomycin, BODIPY™ FL Conjugate - bacteria for fluorescence microscopy 3 h post infection. Acquired microscopic pictures with 10x magnification were analyzed for the number of infected cells by manually counting the bacteria containing cells and the total number of cells per field of view. Data are presented as mean ± SD of 15 fields of views containing in total 8000 cells from three independent experiments (***p<0.001, students t-test). **(C)** LRSAM1 KO (clone #A4) and control cells (A549 NTC) were infected with *S. aureus* HG001 (MOI 10), cells were lysed at the depicted time-points and the released bacteria were plated on blood agar plates for determination of the colony forming units (CFU). Data are represented as mean ± SD of three replicates. Statistical significance is indicated (*p<0.05, students t-test) **(D)** LRSAM1 KO (clone #A9) and control cells (A549 NTC) were infected with *S. aureus* HG001 (MOI 10), extracellular bacteria were assessed at the depicted time-points via plating on blood agar plates for determination of the colony forming units (CFU). Data are represented as mean ± SD of three replicates **(E)** LRSAM1 KO (clone #A9) and control cells (A549 NTC) were infected with PFA-fixed *S. aureus* HG001-GFP (MOI 10) and analyzed for the number of infected cells by flow cytometry at the depicted time points. One out of two experiments is represented here.


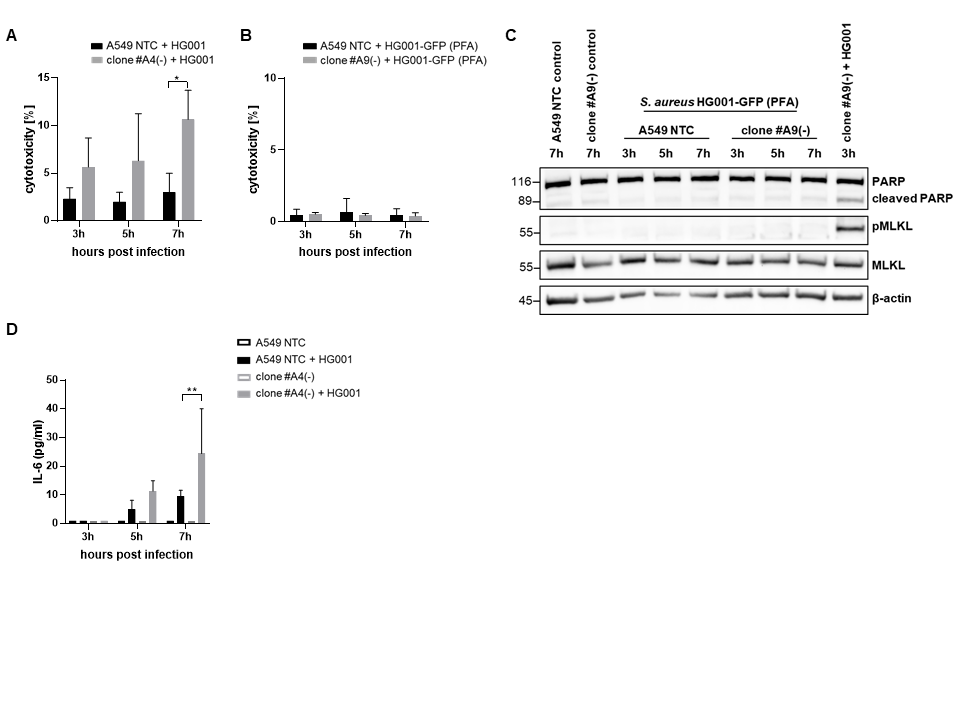


**Supplementary Figure 2.**

**(A)** Host cell death was monitored upon infection with *S. aureus* HG001 (MOI 10) by determining extracellular lactate dehydrogenase (LDH) at the depicted time-points comparing LRSAM1 KO (clone #A4) and control cells (A549 NTC). Data are represented as mean ± SD of three replicates. (*p<0.05, students test) **(B)** Host cell death was monitored upon infection with PFA-fixed *S. aureus* HG001-GFP (MOI 10) by determining extracellular lactate dehydrogenase (LDH) at the depicted time-points comparing LRSAM1 KO (clone #A9) and control cells (A549 NTC). Data are represented as mean ± SD of two replicates. **(C)** LRSAM1 KO (clone #A9) and control cells (A549 NTC) were infected with PFA-fixed *S. aureus* HG001-GFP (MOI 10) for the depicted time-points and subsequently analyzed for PARP cleavage and phosphorylation of MLKL and total MLKL by immunoblotting with β-actin as loading control. A sample of LRSAM1 KO (clone #A9) cells infected for 3 h with *S. aureus* HG001 (MOI 10) was added to the immunoblot for comparison, n = 2. **(D)** Secretion of IL-6 was determined by ELISA for the depicted time points in cell culture supernatants of LRSAM1 KO (clone #A4) and control cells (A549 NTC) during infection with *S. aureus* HG001 (MOI 10). Data are represented as mean ± SD of three replicates. Statistical significance is indicated (**p<0.01, students t-test)


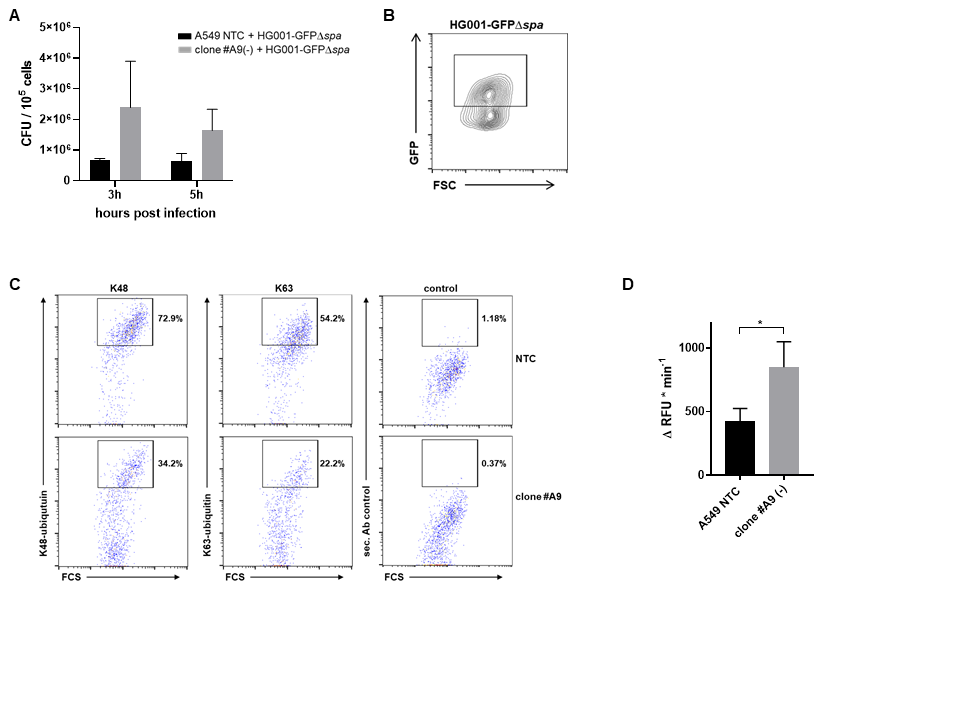


**Supplementary Figure 3.**

**(A)** LRSAM1 KO (clone #A9) and control cells (A549 NTC) were infected with *S. aureus* HG001-GFP∆*spa* (MOI 10), cells were lysed at the depicted time-points and the released bacteria were plated on blood agar plates for determination of the colony forming units (CFU). Data are represented as mean ± SD of two replicates **(B)** Gating example for GFP^+^ intracellular HG001-GFP∆*spa*. **(C)** LRSAM1 KO (clone #A9) and control cells (A549 NTC) were infected with *S. aureus* HG001-GFP∆*spa* (MOI 10) and lysed 3 h post infection. Released intracellular bacteria were analyzed for ubiquitin chain formation using K48- and K63-specific antibodies and subsequent staining with anti-rabbit-Alexa-647 secondary antibody, n = 2. The background staining of the secondary antibody was evaluated using the secondary antibody alone as a control, with no primary antibodies present. **(C)** Cell lysates from LRSAM1 KO (clone #A9) and control cells (A549 NTC) infected with *S. aureus* HG001 (MOI 10) for 3h were assessed for cathepsin B activity using fluorogenic substrate RR-AMC, Data are represented as mean ± SD of three replicates. (*p<0.05, students test)
